# Clinical and Comorbidity Profile of Clinically Diagnosed Gout in Two Tertiary Hospitals in Dubai, United Arab Emirates: A Retrospective Observational Study

**DOI:** 10.7759/cureus.104780

**Published:** 2026-03-06

**Authors:** Saud Abdullah, Mohannad Ali, Shaikha S AlMadhloum AlSuwaidi, Ahmed Negm, Manal Alzaabi, Momina Malik, Khalid Ali

**Affiliations:** 1 Internal Medicine, Dubai Health, Dubai, ARE; 2 Rheumatology, Dubai Health, Dubai, ARE; 3 College of Medicine, Mohammed Bin Rashid University, Dubai, ARE; 4 College of Medicine, University of Sharjah, Sharjah, ARE

**Keywords:** comorbidities, gout, hyperuricemia, prevalence, united arab emirates

## Abstract

Introduction: Gout is an inflammatory arthritis caused by hyperuricemia and is associated with substantial metabolic comorbidity. Despite increasing global recognition, epidemiological and clinical data on gout in the United Arab Emirates (UAE) remain limited. This study aimed to determine the hospital-based prevalence and characterize the demographic characteristics, comorbidities, and treatment patterns of patients with gout in two major tertiary care hospitals in Dubai, UAE.

Methods: This retrospective observational study was conducted at Rashid Hospital and Dubai Hospital between March 2025 and September 2025. Adult patients with a documented clinical diagnosis of gout were identified through electronic medical records. Demographic characteristics, body mass index (BMI), hyperuricemia status, comorbidities, and urate-lowering therapy use were collected. Hospital prevalence was calculated using the total number of unique adult patient records during the study period. Descriptive statistical analysis was performed.

Results: A total of 654 patients with gout were identified among 142,682 adult records, corresponding to a hospital prevalence of 0.46%. The mean age was 54 years, and most patients were male (74.6%, n = 488). Obesity was highly prevalent, affecting 61.1% (n = 397) of patients, and hyperuricemia was present in 94.1% (n = 601). Comorbidities, including hypertension (59.7%, n = 391), diabetes mellitus (42.5%, n = 278), chronic kidney disease (23.5%, n = 154), and coronary artery disease (14%, n = 92), were common, with a higher burden observed among female patients and those aged ≥50 years. Allopurinol was the most frequently prescribed urate-lowering therapy (84.6%, n = 542), followed by febuxostat (9.2%, n = 59).

Conclusion: Gout was associated with a high burden of comorbidities. Obesity was the most common comorbidity, followed by hypertension, hyperlipidemia, and diabetes mellitus, and finally, chronic kidney disease and cardiovascular disease. Allopurinol was the most commonly prescribed therapy. These findings highlight gout as an important metabolic condition in the UAE and emphasize the need for comprehensive management and further population-based studies to better define its epidemiology and clinical burden.

## Introduction

Gout is an inflammatory disease causing arthritis due to the deposition of monosodium urate crystals in the joints and periarticular tissues due to sustained hyperuricemia [1]. Hyperuricemia can result from overproduction, underexcretion, or both of urate in the circulation. This can be affected by multiple factors, including genetic, dietary, renal disease, obesity, and hypertension, among many others [2].

Epidemiological data suggest that the prevalence of gout is more common in developed countries, noting that it has been increasing in both developed and developing countries over the years [2].

Despite this growing recognition of gout, data are limited regarding the epidemiology of gout in the Middle East [1]. A study conducted in 2016 in Dubai, United Arab Emirates (UAE), concluded that, among other musculoskeletal diseases, gout represents 0.1% prevalence in Dubai’s local population [3]. Moreover, the UAE has among the highest regional rates of obesity and diabetes, with a recent study published in 2025 showing that an estimate of 28% of the population is living with obesity [4].

Hospital-based studies, particularly in large tertiary hospitals, tend to provide valuable insight into disease characteristics, daily burden, associated risk factors, and treatments.

This study aimed to describe the demographic characteristics, metabolic profile, comorbidities, and urate-lowering therapy use among patients with clinically diagnosed gout attending two major tertiary hospitals in Dubai, UAE.

## Materials and methods

Study design and setting

This retrospective observational study was conducted at two tertiary care hospitals under Dubai Health: Rashid Hospital and Dubai Hospital. The study was designed to determine the prevalence of clinically diagnosed gout in both hospitals and to describe the demographic characteristics, comorbidities, and lifestyle factors among affected adult patients. Electronic medical records were reviewed using the electronic medical record system (EPIC) over the study period from March 2025 to September 2025.

Study participants

All adult patients aged 18 years and older with a documented clinical diagnosis of gout in their electronic medical records were included in the study. The diagnosis of gout was primarily based on diagnostic coding consistent with gout in the EPIC problem list or discharge diagnosis, supported by documentation in clinical notes by the treating physician. Patients with duplicate records or an unclear diagnosis were excluded from the analysis.

Sample size

As the study aimed to describe the prevalence of gout in both hospitals, no formal sample size calculation was performed. A pragmatic sampling approach was therefore adopted, and all patients with a documented diagnosis of gout in the electronic medical records during the study period were included. A total of 142,682 unique adult records were identified over the six-month study period, of which 654 patients met the inclusion criteria and were included in the final analysis after data cleaning.

Data collection

Clinical data were retrospectively collected from the institutional EPIC and entered into a structured data collection sheet for analysis. Extracted variables included demographic characteristics (age, sex, nationality, and hospital site), clinical characteristics, comorbid conditions, body mass index (BMI), and medication history. Comorbidities assessed included hypertension, type 2 diabetes mellitus, hyperlipidemia, chronic kidney disease, coronary artery disease, heart failure, ischemic stroke, peripheral artery disease, and hypothyroidism. These conditions were defined based on documented diagnoses in the medical records or diagnostic coding.

Gout-specific variables included documentation of hyperuricemia and the use of urate-lowering therapies. Hyperuricemia was defined according to internationally accepted laboratory thresholds (serum uric acid >7 mg/dL in men and >6 mg/dL in women). Body mass index was calculated for each patient and categorized as normal (<25 kg/m²), overweight (25-29.9 kg/m²), or obese (≥30 kg/m²).

Lifestyle variables, including smoking status, alcohol intake, physical activity, and dietary habits, were recorded when available in the electronic medical records. Missing data were not imputed, and analyses were performed using available recorded values for each variable.

Statistical analysis

Data analysis was performed using R (v4.5.2; R Core Team 2025) in RStudio (Posit, Boston, MA) software for macOS (version 2025.09.2+418). All extracted data were anonymized prior to analysis. Owing to the retrospective observational design of the study, analyses were primarily descriptive. Continuous variables were presented as means with standard deviations or medians with interquartile ranges, as appropriate. Categorical variables were summarized as frequencies and percentages.

Hospital-based prevalence was calculated by dividing the number of confirmed gout cases by the total number of unique adult patient records during the study period. Confidence intervals were reported when appropriate, with 95% correspondence.

Subgroup analyses were conducted according to sex (male versus female), nationality (UAE versus non-UAE), and age group (≥50 years versus <50 years). Descriptive comparisons were performed across these groups. Chi-squares were used, and a p-value of less than 0.05 was considered statistically significant.

Ethical considerations

Ethical approval was obtained from the Mohammed Bin Rashid University Institutional Review Board (IRB No. MBRU-IRB-2025-513). Patient confidentiality was maintained in accordance with institutional standards.

## Results

Demographic and clinical characteristics

A total of 654 patients with clinically diagnosed gout were included in the study. The hospital-based prevalence of gout across both institutions was 0.46% (654/142,682). The mean age of the study population was 54 years, and most patients were male (74.6%, n = 488). More than half of the cohort were UAE nationals (57.3%, n = 375). Obesity was highly prevalent, with 61.1% (n = 397) of patients classified as obese, and hyperuricemia was present in the majority of patients (94.1%, n = 601). Hypertension was the most common observed comorbidity (59.7%, n = 391), followed by diabetes mellitus (42.5%, n = 278), chronic kidney disease (23.5%, n = 154), and coronary artery disease (14%, n = 92). Baseline demographic and clinical characteristics are summarized in Table 1.

**Table 1:** Participants' baseline demographic and clinical characteristics ^1^ Percentages are calculated based on valid N. Values are presented as N (%). CI = confidence interval, UAE = United Arab Emirates

Characteristic	Valid N	N (%)^1^	Mean	95% CI
Age in years	654	654 (100%)	54	53-56
Gender				
Male	654	488 (74.6%)	-	
Female		166 (25.4%)		
Nationality				
UAE	654	375 (57.3%)	-	
Philippines		57 (8.7%)		
Egypt		29 (4.4%)		
India		24 (3.7%)		
Oman		23 (3.5%)		
Other nationalities		146 (22.3%)		
BMI				
Normal/underweight (BMI <25)	650	72 (11.1%)	-	
Overweight (BMI 25 – 29.9)		181 (27.8%)		
Obese (BMI > 30)		397 (61.1%)		
Hyperuricemia	639	601 (94.1%)		

Comorbidity profile by demographic groups

Data were stratified by sex, age group, and nationality.

Hypertension was present in 69.3% (n = 115) of females and 56.6% (n = 276) of males (χ² = 6.875, p = 0.009). Type 2 diabetes mellitus was observed in 54.8% (n = 91) of females compared with 38.3% (n = 187) of males (χ² = 12.236, p < 0.001). Chronic kidney disease was identified in 34.9% (n = 58) of females and 19.7% (n = 96) of males (χ² = 15.399, p < 0.001).

When stratified by age group, hypertension was more prevalent among patients aged ≥50 years (73.6%, n = 290) compared with those aged <50 years (38.8%, n = 101) (χ² = 73.906, p < 0.001). Similarly, type 2 diabetes mellitus was present in 56.1% (n = 221) of patients aged ≥50 years compared with 21.9% (n = 57) of those aged <50 years (χ² = 71.766, p < 0.001).

By nationality, type 2 diabetes mellitus was more common among UAE nationals (46.4%, n = 174) than non-UAE patients (37.3%, n = 104) (χ² = 3.941, p = 0.047).

These subgroup comparisons are summarized in Table 2.

**Table 2:** Prevalence of comorbidities by sex, nationality, and age group among patients with gout (n = 654) ^1 ^Values are presented as N (%). P-value was calculated using Pearson’s Chi-squared test. A p-value of < 0.05 is considered significant. Percentages are column-wise. UAE = United Arab Emirates

Characteristic	Group 1			Group 2			Group 3		
	Male N = 488^1^	Female N = 166^1^	Chi-square test	UAE N = 375^1^	Non-UAE N = 279^1^	Chi-square test	Age ≥ 50 years N = 394^1^	Age < 50 years N = 260^1^	Chi-square test
Hypertension (HTN)	276 (56.6%)	115 (69.3%)	X^2^ = 6.875, p = 0.009	235 (62.7%)	156 (55.9%)	X^2 ^= 1.681, p = 0.2	290 (73.6%)	101 (38.8%)	X^2 ^= 73.906, p < 0.001
Type 2 diabetes (T2DM)	187 (38.3%)	91 (54.8%)	X^2 ^= 12.236, p < 0.001	174 (46.4%)	104 (37.3%)	X^2 ^= 3.941, p = 0.047	221 (56.1%)	57 (21.9%)	X^2 ^= 71.766, p < 0.001
Hyperlipidemia	276 (56.6%)	117 (70.5%)	X^2 ^= 8.630, p = 0.003	236 (62.9%)	157 (56.3%)	X^2 ^= 1.445, p = 0.2	278 (70.6%)	115 (44.2%)	X^2 ^= 43.016, p < 0.001
Coronary artery disease (CAD)	68 (13.9%)	24 (14.5%)	X^2 ^= 0.014, p > 0.9	54 (14.4%)	38 (13.6%)	X^2 ^= 0.011, p= > 0.9	82 (20.8%)	10 (3.8%)	X^2 ^= 36.079, p < 0.001
Chronic kidney disease (CKD)	96 (19.7%)	58 (34.9%)	X^2 ^= 15.399, p < 0.001	96 (25.6%)	58 (20.8%)	X^2 ^= 1.621, p = 0.2	126 (32.0%)	28 (10.8%)	X^2 ^= 37.793, p < 0.001
Heart failure (HF)	32 (6.6%)	19 (11.4%)	X^2 ^= 3.873, p = 0.049	29 (7.7%)	22 (7.9%)	X^2 ^= 0.044, p = 0.8	47 (11.9%)	4 (1.5%)	X^2 ^= 22.921, p < 0.001
Ischemic stroke	23 (4.7%)	11 (6.6%)	X^2 ^= 0.821, p = 0.4	16 (4.3%)	18 (6.5%)	X^2 ^= 1.829, p = 0.2	31 (7.9%)	3 (1.2%)	X^2 ^= 13.990, p < 0.001
Peripheral artery disease (PAD)	15 (3.1%)	5 (3.0%)	X^2 ^= 0.005, p > 0.9	12 (3.2%)	8 (2.9%)	X^2 ^= 0.026, p = 0.9	19 (4.8%)	1 (0.4%)	X^2 ^= 10.157, p = 0.001
Hypothyroidism	18 (3.7%)	37 (22.3%)	X^2 ^= 54.441, p < 0.001	35 (9.3%)	20 (7.2%)	X^2 ^= 0.716, p = 0.4	37 (9.4%)	18 (6.9%)	X^2 ^= 1.074, p = 0.3

Distribution of urate-lowering therapy

Urate-lowering therapy use is shown in Table 3. Allopurinol (84.6%, n = 542) was the most commonly prescribed agent, while febuxostat (9.2%, n = 59) was used in a small proportion of patients. No patients received probenecid or pegloticase.

**Table 3:** Distribution of urate-lowering therapy ^1^ Percentages are calculated based on Valid N. Values are presented as N (%).

Urate-lowering therapy	Valid N	N (%)^1 ^
Allopurinol	641	542 (84.6%)
Febuxostat	641	59 (9.2%)
Probenecid	641	0 (0%)
Pegloticase	641	0 (0%)

Lifestyle characteristics 

Lifestyle-related variables were inconsistently documented in the electronic medical records. Smoking status was available for 594 patients, among whom 26.3% (n = 156) were current or former smokers. Alcohol consumption was documented in 507 patients, with 7.7% (n = 39) reporting alcohol use. Physical activity data were available for only 189 patients, of whom 29.1% (n = 55) were classified as physically active. Dietary compliance was documented in 124 patients, with 52.4% (n =65) reported as compliant. Consequently, the substantial proportion of missing data limited further detailed analysis of lifestyle-related factors.

## Discussion

Our retrospective descriptive study provides insight into the prevalence and clinical characteristics of gout among adult patients in the two main tertiary care centers in Dubai. The estimated hospital prevalence was 0.46%, and given the limited local and regional epidemiological data, this finding contributes meaningfully to the existing literature. The prevalence observed in our study is higher than the 0.1% prevalence reported in Dubai in 2016 [3], but lower than the UAE point prevalence of 0.66% reported in a 2019 regional analysis [1]. This places our findings between previously published estimates. These differences may be explained by methodological variation, as population-based studies are more representative of the general population, whereas hospital-based studies are influenced by healthcare access, referral patterns, and case ascertainment. Nevertheless, our findings remain broadly consistent with local and regional data and reinforce that gout represents a significant clinical burden in tertiary care settings. Compared with smaller studies from Saudi Arabia and Bahrain, which reported variable prevalence and smaller sample sizes, our cohort represents one of the larger hospital-based gout populations described in the UAE to date [5,6].

Comorbidities were highly prevalent in our cohort, particularly obesity. A total of 61.1% (n = 397) of patients had a BMI ≥30 kg/m², which is more than double the reported obesity prevalence of 28% in the general UAE population [4]. This highlights the strong association between obesity and gout. Previous studies have demonstrated that for every 5 kg/m² increase in BMI, the risk of developing gout increases by approximately 55%. The underlying mechanisms are multifactorial. Excess adipose tissue increases purine metabolism and urate production through increased activity of xanthine oxidase, the enzyme responsible for converting purines into uric acid, while obesity is also associated with chronic low-grade inflammation, both of which contribute to hyperuricemia [7]. These findings underscore the important role of obesity in gout pathogenesis and emphasize the importance of weight management in disease prevention and long-term control.

Gout predominantly affected men in our cohort, accounting for 74.6% (n = 488) of patients, which is consistent with prior literature demonstrating male predominance [2,8]. This difference is partly attributed to the effects of estrogen, which promotes renal urate excretion in premenopausal women, with gout prevalence increasing after menopause [9]. Despite representing a smaller proportion of the cohort, female patients had a higher prevalence of hypertension, type 2 diabetes mellitus, chronic kidney disease, coronary artery disease, heart failure, and hypothyroidism compared with male patients, consistent with previous reports of a higher cardiometabolic burden among women with gout [10].

Older patients, particularly those aged ≥50 years, had higher rates of hypertension, diabetes, chronic kidney disease, and cardiovascular disease, consistent with established evidence that gout and its associated comorbidities increase with advancing age [2,8]. Hyperuricemia was present in 94.1% (n = 601) of patients, reinforcing its central role in gout pathogenesis and its association with disease progression and comorbid conditions [2,11,12]. These findings support the understanding of gout as part of a broader cardiometabolic disease spectrum rather than an isolated inflammatory condition [1,2,7].

Allopurinol was the most commonly prescribed urate-lowering therapy (84.6%, n = 542), followed by febuxostat (9.2%, n = 59), consistent with guideline recommendations supporting allopurinol as first-line treatment [13]. Only xanthine oxidase inhibitors were used in this cohort; uricase and uricosuric agents were not available at our institutions. The lower use of febuxostat reflects institutional prescribing policies, as it required special approval for prescription, limiting its routine use. 

Lifestyle-related variables, including diet, alcohol use, smoking, and physical activity, were poorly documented in the electronic medical records, limiting the interpretation of these factors. This may reflect underreporting or incomplete documentation and should be considered when interpreting the findings.

This study has several strengths. It represents one of the larger hospital-based analyses of gout in the UAE and includes data from two major tertiary care centers. The comprehensive evaluation of comorbidities and treatment patterns provides valuable real-world insight into the clinical profile and management of gout in this region.

However, several limitations should be acknowledged. The retrospective design limits causal inference, and as a hospital-based study, the findings may not be generalizable to the broader population, particularly those managed in primary care. Reliance on electronic medical records may also result in incomplete data capture.

Future prospective, population-based studies are needed to better define the epidemiology and clinical burden of gout in the UAE and the wider Middle East and North Africa region, where primary epidemiological data remain limited [1]. Improved clinical documentation and standardized data collection will be important to strengthen regional evidence and guide clinical practice and health policy.

## Conclusions

In conclusion, this two-center retrospective study provides contemporary hospital-based insights into the clinical characteristics, metabolic comorbidities, and urate-lowering therapy patterns of patients with gout in Dubai. Gout was associated with a substantial burden of obesity, cardiometabolic disease, and chronic kidney disease, with higher comorbidity prevalence observed among female patients and those aged 50 years and older. Hyperuricemia was almost universal, and allopurinol was the predominant urate-lowering therapy, consistent with current international treatment recommendations.

The limited documentation of lifestyle-related factors underscores a critical gap in routine practice, which hinders comprehensive assessment of modifiable risk factors and treatment effectiveness. Overall, these findings reinforce the need for integrated, multidisciplinary approaches to gout management that extend beyond urate control to address metabolic and cardiovascular comorbidities. Further prospective and population-based studies are required to better define the epidemiology of gout in the UAE and support targeted prevention and management strategies.

## References

[1] Amiri F, Kolahi AA, Nejadghaderi SA (2023). The burden of gout and its attributable risk factors in the Middle East and North Africa region, 1990 to 2019. J Rheumatol.

[2] Asghari KM, Zahmatyar M, Seyedi F (2024). Gout: global epidemiology, risk factors, comorbidities and complications: a narrative review. BMC Musculoskelet Disord.

[3] Al Saleh J, Sayed ME, Monsef N, Darwish E (2016). The prevalence and the determinants of musculoskeletal diseases in Emiratis attending primary health care clinics in Dubai. Oman Med J.

[4] Abdelgadir E, Rashid F, Bashier A, Zidan M, McGowan B, Alawadi F (2025). Prevalence of overweight and obesity in adults from the Middle East: a large-scale population-based study. Diabetes Obes Metab.

[5] Khormi AA, Jain S, Basalem AA (2024). Clinical profile and patient-doctor communication in gout patients: a feasibility study. Bahrain Med Bull.

[6] Alshahrani JA, Saleh Alzahrani SA, Ali AlGhamdi OS (2024). Gouty arthritis across ages: understanding disease patterns and predictors. Cureus.

[7] Timsans J, Palomäki A, Kauppi M (2024). Gout and hyperuricemia: a narrative review of their comorbidities and clinical implications. J Clin Med.

[8] Kuo CF, Grainge MJ, Zhang W, Doherty M (2015). Global epidemiology of gout: prevalence, incidence and risk factors. Nat Rev Rheumatol.

[9] Hak AE, Curhan GC, Grodstein F, Choi HK (2010). Menopause, postmenopausal hormone use and risk of incident gout. Ann Rheum Dis.

[10] Roddy E, Zhang W, Doherty M (2007). The changing epidemiology of gout. Nat Clin Pract Rheumatol.

[11] Dalbeth N, Merriman TR, Stamp LK (2016). Gout. Lancet.

[12] Borghi C, Agabiti-Rosei E, Johnson RJ (2020). Hyperuricaemia and gout in cardiovascular, metabolic and kidney disease. Eur J Intern Med.

[13] FitzGerald JD, Dalbeth N, Mikuls T (2020). 2020 American College of Rheumatology guideline for the management of gout. Arthritis Care Res (Hoboken).

